# Perceived Risk Modifies the Effect of HIV Knowledge on Sexual Risk Behaviors

**DOI:** 10.3389/fpubh.2013.00033

**Published:** 2013-09-30

**Authors:** Gholamhossein Noroozinejad, Mosaieb Yarmohmmadi Vasel, Fatemeh Bazrafkan, Mahmoud Sehat, Majid Rezazadeh, Khodabakhsh Ahmadi

**Affiliations:** ^1^Faculty of Medicine, Qom University of Medical Sciences, Qom, Iran; ^2^Department of Psychology, Bu-Ali Sina University, Hamedan, Iran; ^3^Universal Network for Health Information Dissemination and Exchange (UNHIDE), Tehran, Iran; ^4^Medicine and Health Promotion Institute, Tehran, Iran; ^5^AIDS Prevention and Control Committee, Welfare Organization State, Tehran, Iran; ^6^Behavioral Sciences Research Center, Baqiyatallah Medical Sciences University, Tehran, Iran

**Keywords:** HIV knowledge, perceived risk, sexual risk behavior, moderator, interaction effect

## Abstract

**Background:** There is a large controversy in the literature about the inter-relations between perceived risk, knowledge, and risk behavior in different settings, and people at HIV risk are not an exception.

**Aim:** To assess additive and multiplicative effect of perceived HIV risk and HIV knowledge on sexual risk behavior of Injecting Drug Users (IDUs).

**Method:** We enrolled 162 street based IDUs to this analysis. Data came from a national survey of IDUs in Iran, with a cross sectional design. Socio-demographics (employment, education, marital status), HIV knowledge, perceived HIV risk, and four different sexual risk behavior were registered. In the first step, using spearman test, the association of HIV knowledge and risk behavior were tested, then possible moderating effect of perceived HIV risk on this association was determined.

**Results:** Although among IDUs with low perceived HIV risk, HIV knowledge was negatively associated with sexual risk behavior (*P* < 0.05 for all), this association was not significant among IDUs with high perceived HIV risk (*P* > 0.05 for all). Thus perceived HIV risk moderated the association between HIV knowledge and sexual risk behavior.

**Conclusion:** Perceived risk should be taken into consideration when studying the effect of HIV knowledge on sexual risk behavior of IDUs. Findings may help us better understand negative effects of fear arousing interventions as a part of HIV prevention media campaigns.

## Introduction

Although some studies don’t agree with the statement that a high level of knowledge is important in obtaining modifications in high-risk behavior (1), other studies stress the relevance of information in achieving effective control over behavior (2, 3).

Health Belief Model (HBM) has provided important insights to predict human behaviors. A recurrent theme in HBM theory is the role of perception about risk and benefits associated with a behavior as an antecedent of behavior (1, 4–8). It is possible, however, that the lack of consistent findings regarding knowledge as a significant antecedent of behavior has occurred because an important (factor, influential factor) has been overlooked. According to this model, high perceived risk of harm should encourage people to take action to reduce their risk. Although this implied positive relation between perceived risk and subsequent protective behavior is observed in many empirical studies, it is often weaker than expected (9). Some studies, however, have found no association or even a negative one (1, 10). Our hypothesis is that contradictions observed in the results might be due to ignoring the intensity of perceived risk.

There are considerable research attempting to understand how people perceive risks, the accuracy of their perception of these risk (11, 12), and role of perceived risk as a determinant of behavior or its change (13, 14). There are also some studies that investigate the association between knowledge and behavior (15, 16), however, based on our knowledge, there is not much studies assessing the association between knowledge and behavior by considering level of perceived risk as a possible moderator (17).

Designing public health risk communication programs to persuade people to avoid risk-reducing action requires a detailed knowledge about the pattern of influence of these programs and how can maximize this effect.

## Materials and Methods

### Design and setting

This cross sectional study conducted on 162 injecting drug users (IDUs) sampled from streets in eight provinces located in various different geographical parts of Iran: Tehran, Shiraz, Esfahan, Arak, Ahvaz, Rasht, Mashhad, and Ardebil. The study was conducted by Behavioral Sciences Research Center, Baqiyatallah University of Medical Sciences during 2009.

### Codes of ethics

The study was approved by the ethical review committee of the Baqiyatallah University of medical science. Participation was voluntary, and informed consent was gained from all participants. Purpose of the study was explained to each participant before his or her consent was sought. Participants were also assured of confidentiality and informed about their right to withdraw from the study at any time. Data was gathered anonymously.

### Participants and sampling

Participants were all IDUs recruited from streets in the above listed cities by snowball sampling over a 7 months period in 2009. Every participant who reported positive history of drug injection during his life-time was considered as IDU. The study did not have any exclusion criteria.

### Process

Structured interviews were conducted by university-trained research assistants. Each interview lasted up to 60 min. No financial incentives were offered to the participants.

### Measures

Socio-demographic data (including marital status, educational level, and occupational situation), HIV knowledge (10 items including condom use, sex with a healthy looking person, HIV transmission routs), and perceived HIV risk (5 item Likert scale) was determined.

### Main outcome

Our questionnaire included the following four items to assess sexual risk behavior: During the past 6 months, how many times have you had condom-less sexual relationship with a person who was drug injector? During the past month, with how many people have you had sexual relationship? During the past month, totally how many times have you had condom-less sexual relationship?, and During the past month, how many times had you sex with an Illicit drug user?

### Statistical analysis

Data analysis was conducted in the Statistical Package for the Social Sciences 17 (SPSS Inc., IL, USA) for Windows. According to perceived risk, participants were entered in one of the following groups: high perceived risk (very high/high) and low perceived risk (moderate, low, very low). Spearman’s test was applied to evaluate the correlation between level of knowledge and level of sexual risk behaviors in each stratum. Based on Baron and Kenny a moderator was defined as a variable affecting the direction and/or magnitude of an association between an independent (predictor) and a dependent (outcome) outcome (18). *P* < 0.05 was considered significant.

## Results

From the 162 IDUs recruited to this study, 90 participants were categorized as having low perceived HIV risk, while 72 participants were considered to have high perceived HIV risk. In low perceived risk group, 50% were single, 93% had at least primary education, and 43% were unemployed. Among high perceived HIV risk IDUs, 55% were single, 90% had at least primary education, and 37% were unemployed. There was no significant difference in comparison of socio-economics between these groups (Table 1).

**Table 1:** **Socio-demographic data among 162 Iranian IDUs**.

	Perceived HIV risk			
	High		Low	
	*n*	%	*n*	%
**MARITAL STATUS**				
Single	40	55.6	45	50
Married	27	37.5	45	50
**EDUCATIONAL LEVEL**				
Illiterate	7	9.7	13	14.4
Primary school	18	25	21	23.3
Some secondary school	26	36.1	40	44.4
High school diploma	11	15.3	7	7.8
Associated degree	6	8.3	8	8.9
Bachelor’s degree and higher	2	2.8	1	1.1
**OCCUPATION STATUS**				
Student	0	0	1	1.1
Self-employe	26	36.1	28	31.1
Employe	2	2.8	2	2.2
Retired	0	0	1	1.1
Home maker	3	4.2	6	6.0
Work at private composite	5	6.9	7	7.8
Unemployed	27	37.5	39	43.3

There was no significant difference between the two groups in any of the variables. Chi square.

Among IDUs with high perceived HIV risk, HIV knowledge was not associated with sexual risk behavior (*P* > 0.05 for all correlations). Among IDUs with low perceived HIV risk, high level of HIV knowledge was associated with lower levels of sexual risk behavior (*P* < 0.05 in all correlations) (Table 2).

**Table 2:** **Correlation between HIV knowledge and sexual risk behavior among Iranian IDUs with low versus high perceived HIV risk**.

	Perceived HIV risk			
	High		Low	
	Correlation coefficient	*P* value	Correlation coefficient	*P* value
Number of condom-less sexual relations with a drug injector during the past 6 months	0.232	0.056	0.366	0.005
Number of sexual partners during the previous months	−0.175	0.201	−0.334	0.017
Number of condom-less sexual relations during the past months	−0.156	0.219	0.285	0.03
Number of sexual relations with an illicit drug user during the past months	−0.122	0.353	0.318	0.02

## Discussion

Current study aimed to assess additive and multiplicative effect of perceived risk and HIV knowledge on sexual risk behavior among IDUs. This study showed a multiplicative effect of perceived risk and HIV knowledge on sexual risk behavior of IDUs. That said, higher HIV knowledge had no association with lower sexual risky behaviors in IDUs with high/very high levels of perceived HIV risk, however this association was found among IDUs who had very low to moderate levels of perceived HIV risk.

In this paper, perceived HIV risk was conceptualized as a buffer and HIV knowledge as a protective factor and sexual risk behaviors as outcome. In other words, the relationship between level of HIV knowledge and sexual risk behavior among IDUs may depend on level of perceived risk.

A study by Braithwaite showed no relationship between HIV/AIDS knowledge and generalized sexual risk-taking behaviors (19). The absence of such a relationship was implied that knowledge about risk does not necessarily translate into protective behaviors. These findings were also consistent with a study by Salgado de Snyder et al. (20) demonstrating that while women had information regarding means of HIV/AIDS transmission and ways of prevention, they did not use their knowledge to evaluate, moderate, and substantially change their risk behavior. Although there are other studies in line with the above two studies (21, 22), there are also a few studies showing an association between knowledge and risk behaviors (2, 23).

As previously noted, perceived risk has important and determining role in forming risk behaviors. That means, high perceived HIV risk is known to be associated with less HIV risk behaviors. Several health behavior theories such as HBM (4, 6, 18, 24), theory of reasoned action (25), theory of planned behavior (26), protection Motivation Theory (27), and the AIDS risk reduction model (28) supported this concept. A meta-analysis of 26 studies examining perceived HIV vulnerability and safer sex behaviors has also confirmed this association (1).

According to these models perception about vulnerability can influence precautionary and protective health behaviors. However, studies have shown that this link exists only when preventive behavior is not complex and negative events with extreme threat are absent. In the other words, perception can influence health and risk behaviors, but under special conditions.

Our findings might have implications for HIV AIDS health education. One approach of HIV health education is provision of factual information, which is pointing to adverse possible consequences of risk behaviors to individuals. This approach was applied by several AIDS campaigns in a number of countries such as Switzerland, Netherlands, and Germany. These health educations communicate facts about AIDS risk, and transmission routes of HIV and recommends protective behaviors or abstinence.

Another approach of HIV education tends to rely on fear-arousal as a necessary agent for behavioral change. This approach also tends to emphasis negative consequences of not taking preventive actions. Fear-arousal approach was the key element in the early campaigns in the UK. It has been suggested that a drawback of fear-arousal approach is that high levels of fear may lead to denial and reduce the impact of health education. The fear-drive model assumes a curvilinear relationship between fear-arousal and behavior change. The reason for such pattern is that intensive feelings of anxiety can set off defensive reactions such as a failure to pay attention to the health protective messages, rejection of the communication, or defensive avoidance of anxiety-arousing thoughts (13). Coping strategies is a response often used to protect against anxiety and/worry, and might be activated under condition of high stress or treat denial. Although denial reduces emotional distress, it may also reduce likelihood of preventive actions that are necessary to protect health (17). The result of our study also indicated that among people with high perceived HIV risk, HIV knowledge may fail to reduce risky behaviors. This finding might be explained by the above mechanism.

There are at least two main approaches to reduce the burden of HIV/AIDS in the community; treatment and prevention. Preventive strategies use mass media campaigns, condom distribution, peer-education of prostitutes, voluntary counseling and testing, and diagnosis and treatment of other sexually transmitted diseases (29).

Interventions to prevent HIV/AIDS can be grouped into four main categories, including information based approaches, coping skills acquisition, counseling approaches, and contact with affected groups (30). Community-level interventions have improved HIV risk knowledge, enhanced condom use, and increased perceived HIV risk (31). Although we could not find interventions targeting risk behavior of the populations, that have reported interaction between perceived risk and HIV information (32), theory based HIV interventions will influence behaviors through changing perceptions and attitudes (28, 33–35).

Our findings may have implications for the development and implementation of HIV prevention programs targeting IDUs in Iran. The findings suggest that HIV prevention programs that primarily emphasize fear-arousal AIDS information will not necessarily increase IDUs’ HIV-risk aversion. Although in recent years, policy makers have paid more attention to develop training programs about HIV (36–39), the increasing trend of HIV epidemic shows some problems with these training programs. Although one can not completely deny the current role of media (TV, radio) (39), their role might be increased with a shift in content. Different populations might need benefit from different levels of perceived HIV risk.

Our study has several limitations. Sample size was low. Design was cross sectional. Sampling was not random, thus the results are not generalizable to all Iranian IDUs. Our study did not use standardized inventories to measure HIV knowledge, perceived risk, and sexual behaviors. The study was also limited by only measuring individual level factors (40).

Despite of the limitations, the study may help us better understand the complex interplay between socio-economic factors, knowledge, attitude, and risk behavior (41–46). Unfortunately, we do not know much about the complex relations between cognition, attitude, and risk behaviors among IDUs. We also do not know how the associations between these psychological constructs vary from one population to another, or from one context to the other. We know that sexual behavior is a complex behavior and is under influence of a long list of variables (47–53).

Promotion of health and wellbeing of general population and HIV risk groups needs research based evidence that can be used to design strategies for health promotion. The same is true for prevention of HIV among IDUs and other HIV risk groups (53–58). It is the collective knowledge – derived from research – that enables policy makers and public health practitioners to prevent blood born infections such as HIV and hepatitis in the community (59–69).

## Conclusion

Increasing perceived risk among IDUs does not necessarily result in reduction of risk behaviors. We showed that perceived HIV risk may modify protective effect of HIV knowledge on sexual risk behaviors of IDUs. Further studies, specially using prospective design will be beneficial to understand additive and multiplicative effects of perceived HIV risk and HIV knowledge on HIV risk behaviors. Findings may have implications for media campaigns that use HIV risk communication approach.

## Conflict of Interest Statement

The authors declare that the research was conducted in the absence of any commercial or financial relationships that could be construed as a potential conflict of interest.
